# Application of Post Solid-Phase Oxime Ligation to Fine-Tune Peptide–Protein Interactions

**DOI:** 10.3390/molecules25122807

**Published:** 2020-06-18

**Authors:** Xue Zhi Zhao, Fa Liu, Terrence R. Burke

**Affiliations:** 1 Chemical Biology Laboratory, Center for Cancer Research, National Cancer Institute, National Institutes of Health, Frederick, MD 21702, USA; xuezhi.zhao@nih.gov; 2 Discovery Chemistry, Novo Nordisk Research Center Seattle, Seattle, WA 98109, USA; liufasioc@live.com

**Keywords:** peptidomimetics, peptide oxime ligation, tethered fragment libraries, protein–protein interactions (PPIs), post solid-phase diversification

## Abstract

Protein–protein interactions (PPIs) represent an extremely attractive class of potential new targets for therapeutic intervention; however, the shallow extended character of many PPIs can render developing inhibitors against them as exceptionally difficult. Yet this problem can be made tractable by taking advantage of the fact that large interacting surfaces are often characterized by confined “hot spot” regions, where interactions contribute disproportionately to overall binding energies. Peptides afford valuable starting points for developing PPI inhibitors because of their high degrees of functional diversity and conformational adaptability. Unfortunately, contacts afforded by the 20 natural amino acids may be suboptimal and inefficient for accessing both canonical binding interactions and transient “cryptic” binding pockets. Oxime ligation represents a class of biocompatible “click” chemistry that allows the structural diversity of libraries of aldehydes to be rapidly evaluated within the context of a parent oxime-containing peptide platform. Importantly, oxime ligation represents a form of post solid-phase diversification, which provides a facile and empirical means of identifying unanticipated protein–peptide interactions that may substantially increase binding affinities and selectivity. The current review will focus on the authors’ use of peptide ligation to optimize PPI antagonists directed against several targets, including tumor susceptibility gene 101 (Tsg101), protein tyrosine phosphatases (PTPases) and the polo-like kinase 1 (Plk1). This should provide insights that can be broadly directed against an almost unlimited range of physiologically important PPIs.

## 1. Introduction

There are estimated to be more than 400,000 protein–protein interactions (PPIs) comprising the human “interactome” and collectively, these represent an extremely attractive class of potential new targets for therapeutic intervention against a broad range of diseases, including several cancers [1,2,3]. The shallow, extended character of many PPIs can render developing inhibitors against them as exceptionally challenging. However, large interacting surfaces are often characterized by confined “hot spot” regions that contribute disproportionately to overall binding energies [4,5,6]. Hot spots tend to occur packed in clusters, where they form interaction networks that can act in cooperative fashion to promote overall PPI stability [7]. By tethering together multiple components that simultaneously access several of these hot spots, the problem of single ligands disrupting the congregate PPIs can become more tractable [8]. Because PPIs involve interactions of polypeptide sequences presented by the interacting proteins, peptides are natural starting points for PPI ligand development [9,10]. Yet, there are a number of challenges that must be addressed in using peptides to develop PPI inhibitors. The high degrees of conformational flexibility exhibited by peptides makes the application of secondary conformational constraint a potentially important consideration [11,12,13]. Additionally, although peptides afford high degrees of functional diversity, contacts afforded by the 20 natural amino acids may be suboptimal and inefficient for accessing both canonical binding interactions and transient “cryptic” binding pockets [7].

Application of rapid and efficient strategies that can extend chemical diversity beyond that afforded by the coded amino acids has the potential to significantly enhance the ability of peptides to explore unexpected binding interactions, thereby making the identification of high affinity PPI inhibitor leads more probable. Post solid-phase diversification is particularly appealing, since it eliminates the necessity of individually preparing each peptide by total synthesis. Utilizing azide-alkyne “click” chemistry can be a rapid and easy way to introduce structural diversity [14]. However, it may be limited by its requirement for product purification prior to biological evaluation. On the other hand, oxime ligation represents a class of biocompatible click chemistry, whose products can be subjected directly to biological evaluation without a need for intermediate purification [15]. A collection of aldehydes can be coupled in high-yield fashion with an aminooxy-containing parent precursor peptide to yield stable constructs. This allows the structural diversity of libraries of aldehydes to be rapidly evaluated within the context of the original peptide platform [16]. We have synthesized a variety of orthogonally protected aminooxy-containing amino acid analogs and, using standard Fmoc-based solid-phase protocols, we have introduced these aminooxy handles into peptide sequences directed against several PPI targets [17]. The resulting aminooxy-modified peptides require only a single purification step before being subjected to post-solid-phase diversification by oxime ligation that yields libraries of peptides containing a range of non-coded amino acid residues. We have applied this empirical approach to identify unanticipated interactions that have significantly increased binding affinities and selectivity relative to the parent peptides. In the following, we will present a brief overview of our application of peptide oxime ligation for the preparation of ligands directed at PPI interfaces of several targets, including tumor susceptibility gene 101 (Tsg101), protein tyrosine phosphatases (PTPases) and the polo-like kinase 1 (Plk1).

## 2. Oxime Ligation: General Protocol

**Figure 1:**
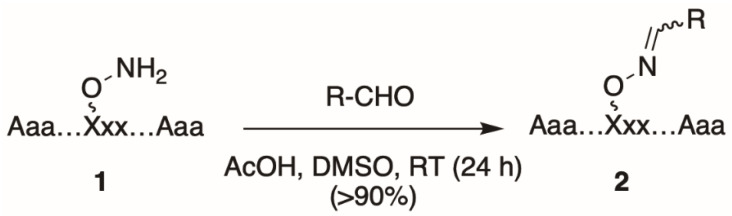
General approach to prepare oxime-diversified peptides.

Oxime tethering reactions require the use of appropriate orthogonally protected aminooxy-containing amino acid derivatives [18,19,20,21,22], which are used to prepare parent aminooxy-containing parent peptides (**1**, Figure 1). Typically, peptide synthesis using these modified amino acids is performed on acid-labile resin under standard Fmoc-based solid-phase protocols. Following completion of peptide synthesis, the aminooxy-containing peptides are cleaved from the resin and the resulting crude peptide mixtures are subjected to preparative HPLC to provide pure stock aminooxy-containing parent peptide (**1**). For post-solid-phase diversification, a solution of **1** in DMSO can be aliquoted into the wells of microtiter plates and into each well is then added a single, unique alkyl or aryl aldehyde in the presence of a slight excess of acetic acid at room temperature (RT) overnight. This results in a single oxime product (**2**) for each well, which is typically of sufficient purity to be used directly for biological testing without a need for further intermediate purification (Figure 1) [17].

## 3. Application to Tsg101-Binding Peptides

**Figure 2:**
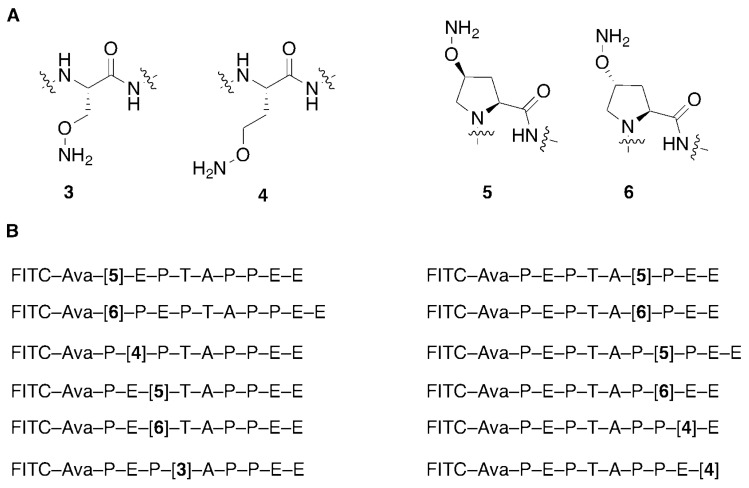
Aminooxy-containing peptides used for Tsg101 oxime library scans. (**A**) Structures of aminooxy-containing residues; (**B**) Tsg101-binding peptides showing the placement of aminooxy residues. (Note: FITC = fluorescein isothiocyanate; Ava = aminovaleric acid).

**Figure 3:**
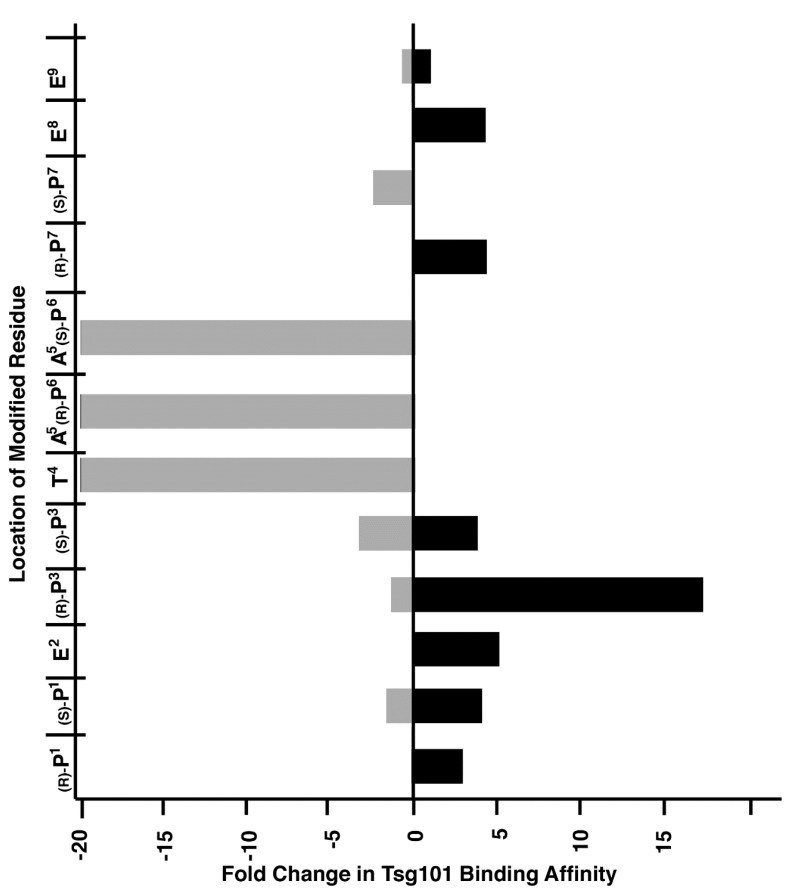
Graphical depiction of maximum effects on Tsg101 binding affinity achieved by modification of each residue of the wild-type sequence. The Y-axis corresponds to the location within the peptide of each aminooxy-containing residue, with the corresponding peptide structures being listed in Figure 2. The range of relative affinities of the resulting oxime-containing peptides at each position is shown on the X-axis. The horizontal bars represent the combined range of affinities over the entire library examined at each position.

We have applied peptide oxime diversification to optimize ligand interactions with a variety of PPI targets. In one instance, we have utilized this approach to develop inhibitors of PPIs associated with viral budding. In the case of HIV-1, budding requires a direct interaction between a Pro-Thr-Ala-Pro (“PTAP”) motif in the viral Gag-p6 protein and the human cellular protein encoded by Tsg101, which is a component of the host endosomal sorting pathway [23]. Inhibition of this Gag-Tsg101 interaction may provide the basis for a new class of AIDS therapies [24]. Tsg101 binding data for a series of PTAP-containing peptides have shown that the p6-derived nonamer sequence “P^1^E^2^P^3^T^4^A^5^P^6^P^7^E^8^E^9^” retains modest binding affinity. In order to probe the nature of the binding interactions of the parent peptide with Tsg101, we conducted a systematic examination of each residue using an oxime library approach [25]. For each position in the fluorescently labeled peptide, we selected aminooxy-containing analogs that approximated the parent residue and performed an oxime scan using a mixed library of aliphatic and aryl aldehydes (Figure 2). The structures of these aldehydes were chosen such that a diversity of new peptide–protein interactions were examined at each residue position. Such a single library established a two-dimensional structure–activity relationship profile of the nonamer peptide, where each residue was tethered with a set of structurally diverse fragments systematically probing the respective adjacent areas on the Tsg101 surface. This identified T^4^A^5^P^6^ as residues that were intolerant to modification (Figure 3).

**Figure 4:**
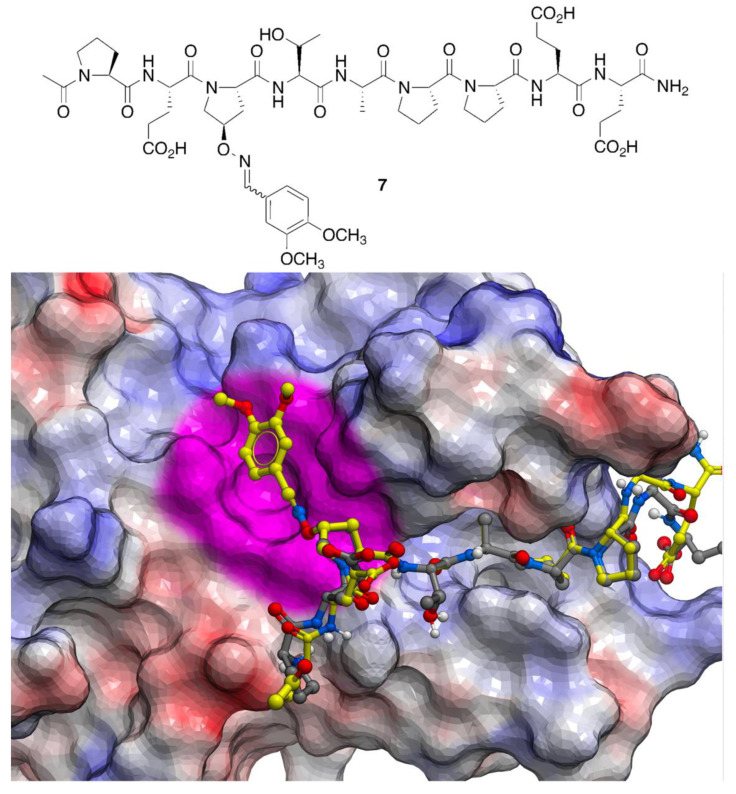
Crystal structures of parent p6-derived PEPTAPPEE bound to Tsg101 (carbons in grey; PDB: 3OBU) showing electrostatic surfaces (blue = positive; red = negative and white = neutral). Structure of peptide **7** (carbons in yellow; PDB: 3P9H) is superimposed showing the location of an unanticipated binding region discovered by the oxime scan (highlighted in magenta).

This positional scan showed that modification of the P^3^ position with aryl-containing oximes, such as 3,4-dimethoxybenzene, could enhance binding affinities from 15- to 20-fold (Figure 4). Subsequently, a crystal structure of 3,4-dimethoxybenzeneoxime-containing peptide **7** bound to Tsg101 protein (Protein Data Bank (PDB) accession code 3P9H) [26] identified binding interactions proximal to the P^3^ residue that were not readily anticipated by interactions of the parent peptide (PDB: 3OBU, Figure 4) [27]. As we reported [26], these interactions are amenable to further optimization.

## 4. Application to Peptide–Protein Interactions of Protein Tyrosine Phosphatases

Protein tyrosine phosphatases (PTPases) dephosphorylate phosphotyrosine (pY) residues within proteins and they participate in signal transduction pathways that regulate pathological processes involved in cancers and infectious diseases. Despite success in the development of small molecules that inhibit kinases as potential therapeutic agents, targeting PTPases by small-molecule inhibitors is hindered by their relatively smooth protein surfaces and shallow catalytic pockets. One approach to potentially improving druggable features of PTPases is to take advantage of interactions with protein features outside the catalytic pocket that may be involved in PPIs. An objective of such work is to develop inhibitors of PPIs that are intrinsic to PTPase catalytic specificities.

**Figure 5:**
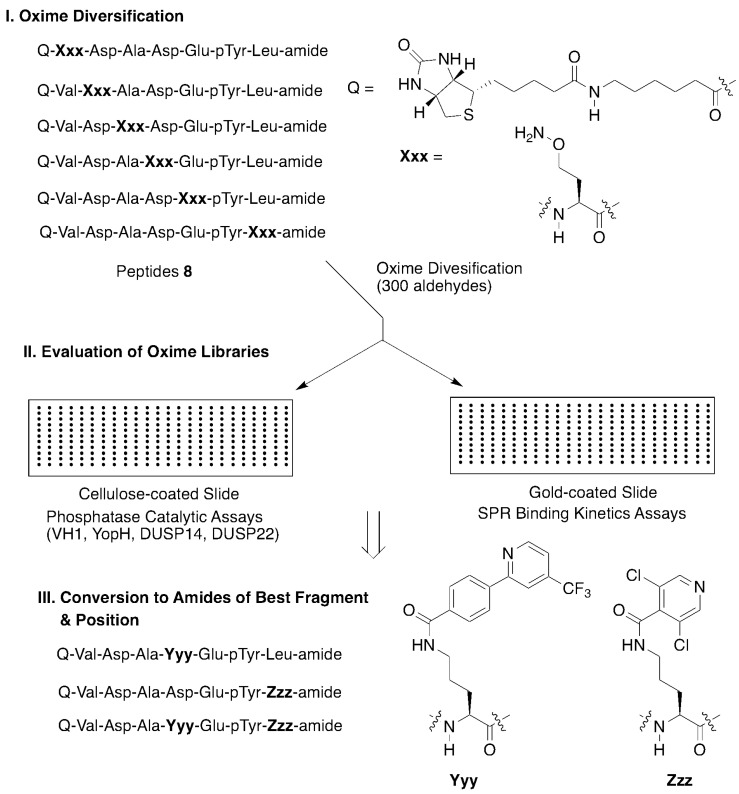
Application of oxime diversification to optimize protein tyrosine phosphatase (PTPase) ligand interactions.

In order to probe for these interactions, we employed an oxime library tethered fragment approach based on the high affinity epidermal growth factor receptor (EGFR)-derived peptide substrate, “VDADEpYL,” which includes the pY992 autophosphorylation site. Keeping the pY constant, an aminooxy-containing residue was sequentially placed at each position of the peptide to yield six oxime-containing peptides (**8**, Figure 5). Solution-phase oxime diversification conducted using a collection of 300 aldehydes yielded a library with 1800 distinct oxime-containing peptides. The libraries were printed in microarrays on nitrocellulose-coated or gold-coated slides and PTPase catalytic activity or binding kinetics were then assessed using a panel of PTPases or surface plasmon resonance (SPR), respectively (Figure 5). Application of this two-step catalytic and SPR-based microarray protocol permitted identification of optimum fragments and positions. Two of the better oximes were converted to their corresponding ornithine amides (**Yyy** and **Zzz**, Figure 5) and ultimately, this led to non-phosphate-containing peptides that showed low micromolar PTPase inhibitory potencies [28].

## 5. Application to Plk1 PBD-Binding Peptides

**Figure 6:**
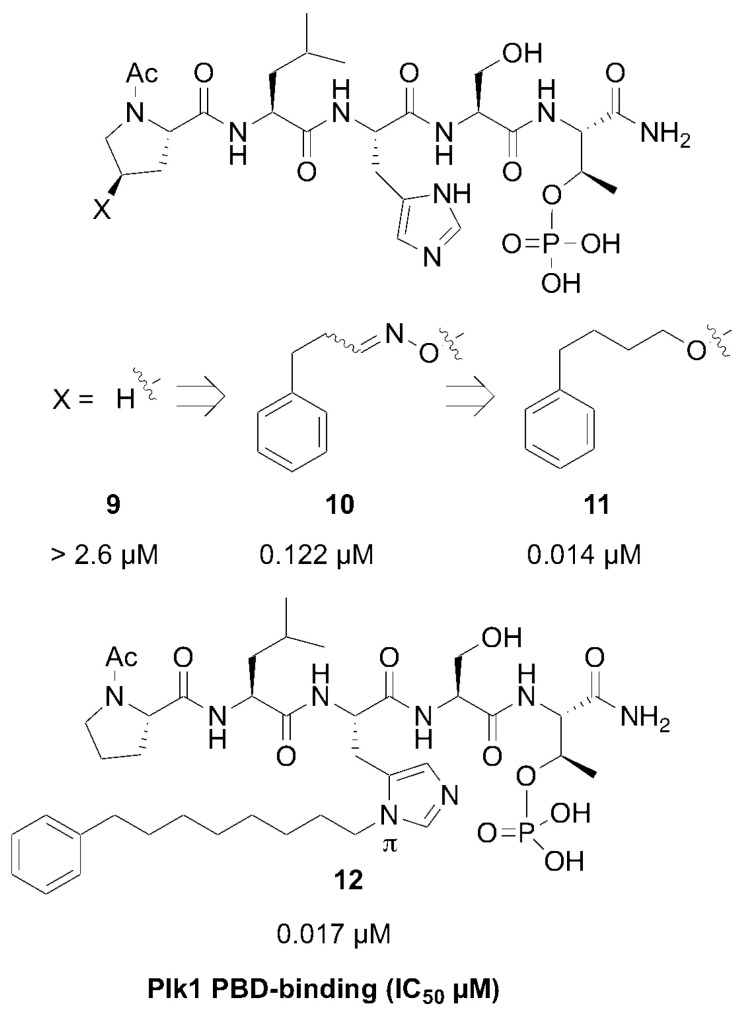
Structures and Plk1 PBD binding affinities of peptides described in the text.

Members of the polo-like subfamily of protein kinases (Plks 1–5) play central roles in cell proliferation, with Plk1 being recognized as a potentially promising anticancer molecular target, because of its ability to promote tumorigenesis in human cells [29]. Plk1 contains both an *N*-terminal catalytic domain and a *C*-terminal polo-box domain (PBD), which functions to engage PPIs in a phosphoserine (pS)/phosphothreonine (pT)-dependent fashion. PPI inhibitors that antagonize PBD-dependent Plk1 function are being pursued as potential therapeutic alternatives to Plk1 kinase inhibitors [30]. One focus of these latter efforts is to optimize the affinities and selectivity of cognate PBD-binding sequences. A widely used starting sequence is the polo-box interacting protein 1-derived 5-mer peptide “PLHSpT” (**9**), which represents a minimal Plk1 PBD-binding sequence having moderate affinity and selectivity relative to two closely related Plk2 and Plk3 PBDs [31]. The crystal structure of Plk1 PBD-bound **9** (PDB: 3HIK) led us to explore structural variations originating from the Pro residue employing an oxime diversification approach. Since biochemical data indicated that this Pro residue is important both for Plk1 PBD-binding affinity and specificity relative to the closely related kinases, Plk2 and Plk3, the pyrrolidine ring of the original residue was maintained and *trans* (4*R*) and *cis* (4*S*) aminooxy substituents were introduced to allow post solid-phase oxime derivatization [32]. The peptides containing oximes formed from 3-phenylpropanal showed the greatest affinity enhancements, with the (4*R*)-isomer (**10**) showing a slight preference, being approximately 20-fold better than the parent peptide **9** (0.122 µM versus 2.6 µM, respectively, Figure 6). Converting the oxime **10** to the corresponding 4-phenylbutyl ether analog, while maintaining the overall chain extension, provided peptide **11**, which at an affinity of 14 nM, represented an additional order-of-magnitude affinity enhancement (Figure 6) [32].

**Figure 7:**
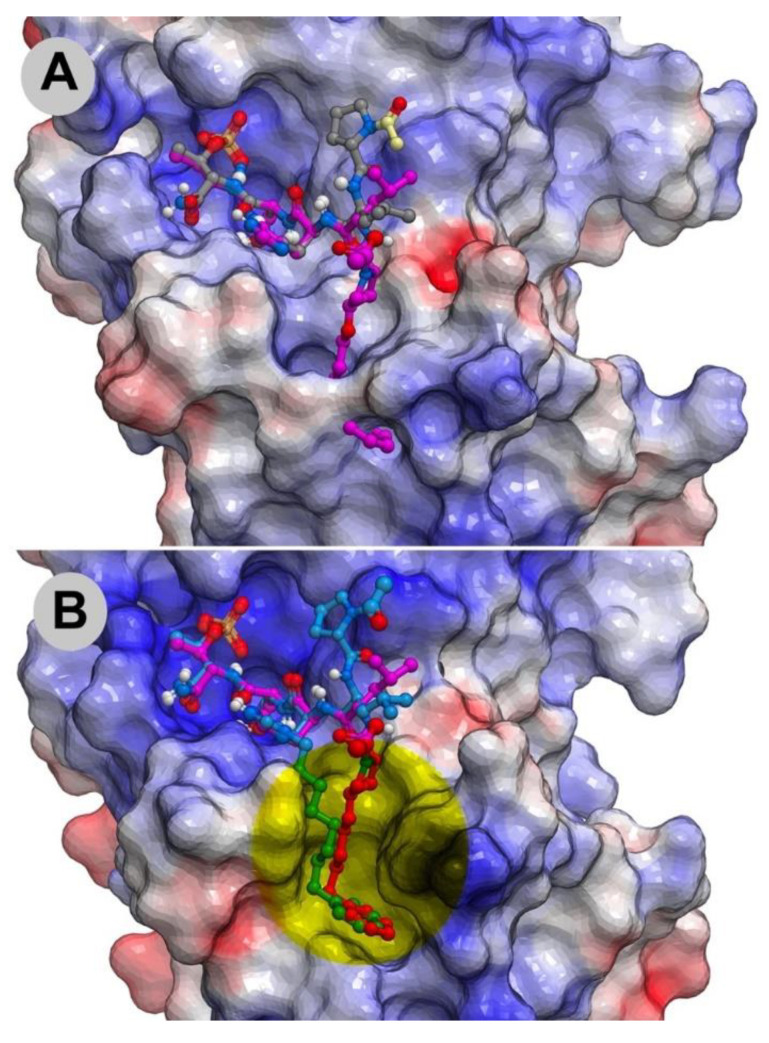
Crystal structures of peptides bound to the Plk1 PBD showing electrostatic surfaces. (**A**) Structure of peptide **11** (carbons in magenta; PDB: 4DFW) superimposed onto the structure of PBD-bound peptide **9** showing the electrostatic surface associated with **9** (carbons in grey; PDB: 3HIK); (**B**) Structure of peptide **12** (PDB: 3RQ7) superimposed onto the structure of PBD-bound **11** with its associated electrostatic surface (PDB: 4DFW) showing similar access of the cryptic pocket in yellow highlight.

The molecular basis for the large affinity enhancement incurred by introduction of the 4-phenylbutyl ether substituent in **11** was not obvious based on the structure of PBD-bound parent peptide **9** (PDB: 3HIK, Figure 7). However, by solving the crystal structure of PBD-bound **11** (PBD: 4DFW), we found that while the “HSpT” residues of **11** were nearly superimposable with those of the parent in the 3HIK structure, differences in the psi angles of the Leu residues placed the adjacent *N*-terminal Pro residues in nearly opposing directions (Figure 7). This situates the (4*R*) phenylbutyloxy group of **11** so that it terminates with its phenyl ring in a “cryptic binding pocket” that is occluded in the crystal structure of **9**. Surprisingly, the interactions of the “-(CH_2_)_4_-Ph” moiety of **11** are highly similar to what are observed with the peptide PLH*SpT, where H* indicates the presence of a “-(CH_2_)_8_Ph” group on the His N3 (π) nitrogen (i.e., His-[*N*(π)-(CH_2_)_8_Ph]) (peptide **12**, PDB: 3RQ7) (Figure 7). Peptide **12** was independently discovered in a serendipitous fashion by the unintended alkylation of the His residue during on-resin Mitsunobu esterification reactions [33]. The approximate 1000-fold enhanced Plk1 PBD-binding affinity of **12** relative to the parent **9** further shows the potential importance of accessing this cryptic pocket.

**Figure 8:**
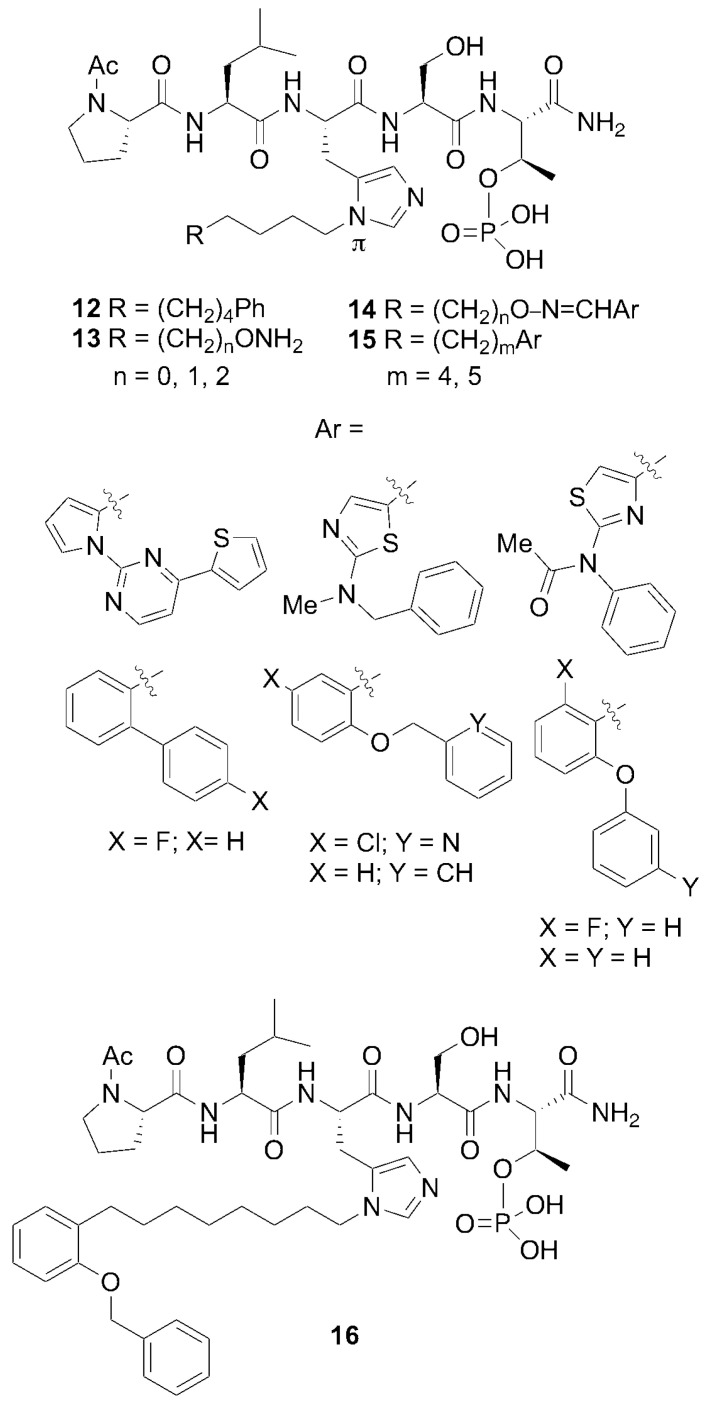
Optimization of the His-[*N*(π)] substituent using an oxime scan approach. The highest-affinity ligands typically had “Ar” groups, consisting of multiple aromatic rings in “bent” configurations. Peptide **15** exhibited approximately four-fold higher affinity than the parent peptide **12**.

In spite of the large contributions that interactions within the cryptic pocket make to overall binding affinity of **12**, little effort had been directed at optimizing these interactions. This was due in large part to the fact that introducing the –(CH_2_)_8_Ph moiety into parent **12** requires a total peptide synthesis using the reagent *N*-Fmoc-His-[*N*(π)-(CH_2_)_8_Ph]-OH [34]. Such a lengthy synthesis makes it laborious to conduct a direct examination of different functionality at the *N*(π)-position. To circumvent this obstacle, we resorted to a tethered fragment methodology that employed oxime ligation [35]. We synthesized an initial set of parent peptides (**13**) having terminal aminooxy groups tethered at various distances from the *N*(π)-position and then performed an oxime scan using a library of over 80 aldehydes. This resulted in a collection of oxime-containing peptides (**14**). By interrogating interactions within the binding pocket in this fashion, we were ultimately able to identify preferred binding motifs, many of which had multiple aromatic rings configured in “bent” orientations (Figure 8). Subsequently, we replaced the oxime linkages with methylene chains (peptide **15**). In this fashion, we eventually arrived at 2-aryl-containing tethered substituents. Some of these analogs, such as peptide **16**, exhibited up to four-fold higher affinities than parent peptide **12** [35]. This is remarkable, since until that point, **12** had been one of the highest-affinity Plk1 PBD-binding ligands yet reported.

**Figure 9:**
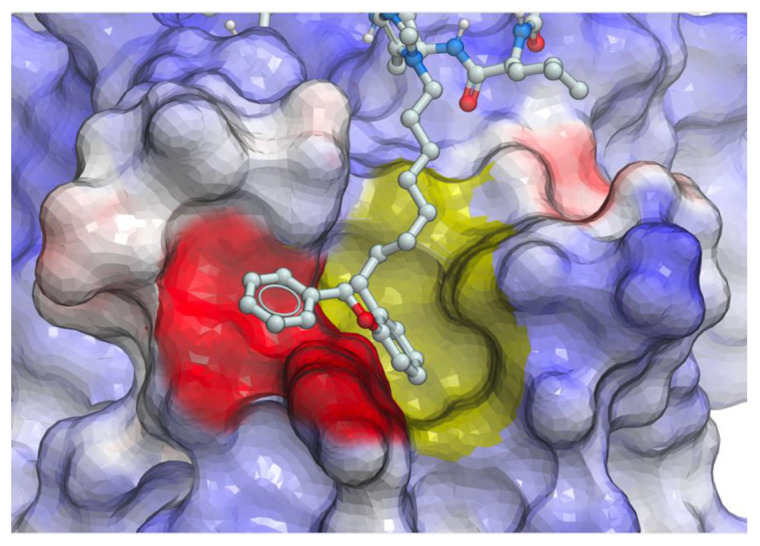
Electrostatic surface of the Plk1 PBD with peptide **16** positioned to show potential interactions with both the cryptic pocket (yellow surface) and proposed “auxiliary” region (red surface).

We hypothesized that the branched bi-aryl ring systems allowed access to a previously unidentified “auxiliary” binding region, proximal to the hydrophobic cryptic pocket (Figure 9).

We also thought that by taking advantage of subtle differences within the proximal auxiliary binding pockets, it might be possible to enhance selectivity for the Plk1 PBD relative to the PBDs of Plk2 and Plk3, since several residues in this region differ among the three PBDs. Accordingly, in further work we were able to arrive at peptides, such as **17**–**19**, which showed up to three orders-of-magnitude selectivity for the PBD of Plk1 relative to Plk3 (Table 1) [36].

## 6. Conclusions

Oxime ligation is an emerging and powerful tool that allows one to interrogate and optimize the interactions of peptides with protein binding partners. Viewed as a “tethered fragment” approach, oxime ligation has distinct advantages over traditional fragment-based screening, where the low binding affinities of fragment libraries present inherent limitations. It combines the power of fragment screening together with affinity enhancement afforded by multi-valency. In this review, we have presented a number of studies, in which we have utilized this approach to optimize peptide–protein interactions. Used effectively, oxime ligation has the potential to profoundly impact the development of PPI inhibitors.

## Figures and Tables

**Table 1:**
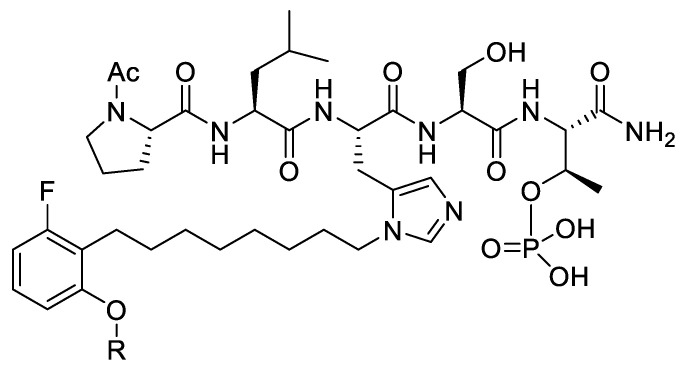
Enhancing Plk1 PBD selectivity by potentially taking advantage of binding interactions in an auxiliary binding region proximal to the cryptic pocket.

No.	R	IC_50_(nM) ^a^		
		Plk1	Plk2	Plk3
**12**	n/a	5	130 (26×) ^b^	280 (56×)
**17**	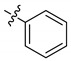	7	290 (44×)	1500 (220×)
**18**	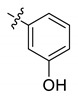	6	3400 (620×)	1900 (345×)
**19**	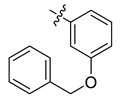	11	3500 (312×)	29,500 (2600×)

^a^ Values determined by fluorescence anisotropy competition assays. ^b^ Fold change relative to Plk1.
